# Au Doping Effect on the Secondary Electron Emission Performance of MgO Films

**DOI:** 10.3390/ma11112104

**Published:** 2018-10-26

**Authors:** Jie Li, Wenbo Hu, Kang Wang, Buyu Gao, Yongdong Li, Shengli Wu, Jintao Zhang, Huiqing Fan

**Affiliations:** 1 Key Laboratory for Physical Electronics and Devices of the Ministry of Education, School of Electronic and Information Engineering, Xi’an Jiaotong University, No. 28, Xianning West Road, Xi’an 710049, China; lijie0814@stu.xjtu.edu.cn (J.L.); wangk5057@stu.xjtu.edu.cn (K.W.); m13689291951@163.com (B.G.); leyond@mail.xjtu.edu.cn (Y.L.); slwu@mail.xjtu.edu.cn (S.W.); zhangjt@mail.xjtu.edu.cn (J.Z.); 2 School of Materials Science and Engineering, Northwestern Polytechnical University, West Youyi Road, Xi’an 710072, China; hqfan@nwpu.edu.cn

**Keywords:** Au-doped MgO films, secondary electron emission, Au doping, first-principle calculation, work function, electrical conductivity

## Abstract

Au-doped MgO films were prepared by reactive sputtering of individual Mg and Au targets, and the Au doping effect on the electron-induced secondary electron emission (SEE) performance was explored by means of surface analysis, first-principle calculation, and electrical characteristic measurement. The results show that the size enlargement of MgO grains and the reduction of surface work functions induced by Au doping are the main reasons for the increase of the SEE coefficient (*δ*). Additionally, the superior SEE degradation property of the Au-doped MgO film under continuous electron bombardment results from the improvement of electrical conductivity. Through the optimization of Au doping concentration (*x*), Au-doped MgO film with an *x* value of 3.0% was found to have the best SEE performance due to its highest SEE coefficient and longest duration of maintaining a relatively high SEE coefficient; its maximum *δ* value reached 11.5—an increase of 32.2% in comparison with the undoped one.

## 1. Introduction

Since its discovery by Campbell, secondary electron emission (SEE) of solid materials has been widely investigated and applied in various electronic information fields such as space navigation, space instruments, night-vision technology, and microanalysis [1,2,3,4]. In these fields, solid materials need to have excellent electron-induced SEE performances for achieving the function of electrical signal amplification. Secondary electron emission generally occurs in three steps: (1) production of internal secondary electrons caused by primary electron bombardment; (2) transport of internal secondary electrons towards the material surface; and (3) escape of internal secondary electrons from the material surface. Insulator materials including beryllium oxide, silicon nitride, diamond, and magnesium oxide (MgO) generally exhibit high SEE coefficients (*δ*) because of their wide band gaps which can help internal secondary electrons reduce collisions with free electrons and have a greater possibility to be emitted from the material surface [5,6,7,8]. Among these materials, MgO has attracted large amounts of attention due to its favorable antisputtering capability besides its high SEE coefficient [9,10,11].

Restricted to the insufficient SEE coefficient at various primary electron energies (*E*_p_) and the surface charging effect under continuous electron bombardment, pure MgO film is not thought to be one of the best materials to meet the demands of high-gain and long-lifetime vacuum electronic devices [12]. As the SEE of a solid material has a close relationship with its surface morphology, electronic structure, and electrical conductivity, many research efforts toward the improvement of the SEE performance, such as substrate temperature rise, Cs termination treatment, and MgO layer thickness adjustment, have been devoted to MgO film materials by effecting changes in these physical properties [13,14,15]. Besides the abovementioned explorations, one of the most promising methods to enhance the SEE performance is the proper doping of other substances in MgO films. For example, Wang et al. reported that CoO doping in MgO films could reduce the surface roughness and thereby result in SEE improvement [16]. Wang et al. prepared MgO/Al_2_O_3_ composite films on silver substrates and achieved superior SEE properties due to both the presence of Al_2_O_3_ having better resistance to electron bombardment than MgO and the residual Al dispersed in the film contributing to good surface charge transfer [17]. Fan et al. proposed MgO/Au, MgO/Ag, and MgO/Ni cermet films grown by sputtering of composite targets, and these films were good secondary electron emitters because the presence of metallic particles in the films mitigated the surface charging effect [18]. These research results showed that proper doping in MgO film could enhance the SEE coefficient. Nevertheless, the SEE performances of these doped MgO films are not sufficient to meet the demands of high-gain and long-lifetime vacuum electronic devices. Thus, further investigations on the SEE mechanism of doped MgO films are needed in order to obtain a better secondary electron emitter.

Since Au has excellent electrical conductivity and chemical stability, the doping of Au in MgO films is a possible method to suppress the surface charging effect to improve the SEE performance of MgO films. Early in 1973, Henrich et al. obtained high-efficiency secondary electron emission from a sputtered MgO/Au cermet system at substrate temperatures ranging from 50 to 350 °C [19]. However, its peak SEE coefficient only reached 8. Thus, the SEE performance of the Au-doped MgO films needs to be improved. Unlike previous reports [19,20], in this work, Au-doped MgO films with low doping concentrations (*x*) were prepared by reactive sputtering of Mg and Au targets at a relatively higher temperature. Furthermore, the effect of Au doping on the surface morphologies, electronic structures, electrical conductivities, and SEE performances of MgO films was analyzed in order to elaborate the SEE mechanism of Au-doped MgO films.

## 2. Experimental Details

Au-doped MgO film samples were prepared by reactive magnetron co-sputtering of high-purity (99.99%) Mg and Au targets on heavy-doped N-type (100) silicon substrates under the conditions of substrate temperature of 500 °C, Ar/O_2_ gas flow rate of 5:1, and deposition chamber pressure of 0.2 Pa. The Au doping concentrations in different MgO film samples were controlled by the sputtering powers of the Au target. In this work, three Au-doped MgO film samples with the doping concentrations of 1.5%, 3.0%, and 4.5% and a pure MgO film sample as a reference were prepared.

For these four samples, their SEE performances were evaluated using a self-designed SEE testing system described in our previous report [13], and their *I*–*V* characteristics were measured using a power device analyzer (B1505A, Agilent, CA, USA). Additionally, their surface morphologies and surface roughnesses were characterized using a scanning electron microscope (SEM, JSM-7000F, JEOL, Ltd., Tokyo, Japan) and an atomic force microscope (AFM, Dimension Icon, Bruker, Billerica, MA, USA), respectively.

As first-principle calculations have been widely applied in the analysis of various physical properties [21], the effect of the Au doping concentration on the electronic structure of the MgO film was analyzed on the basis of first-principle calculations. The band structures and work functions of the undoped and Au-doped MgO crystals were calculated with the Cambridge Serial Total Energy Package simulation program. MgO is an ion-type crystal with a face-centered cubic structure and FM-3M space group, and its lattice parameters and bond angles are a = b = c = 0.421 nm and α = β = γ = 90°, respectively. A 2 × 2 × 2 supercell containing 32 oxygen atoms and 32 metal atoms was adopted for pure MgO, and Au-doped MgO supercells were obtained by replacing several Mg atoms with the same number of Au atoms. Three Au-doped MgO supercells with Au atom numbers of 1, 2, and 3 were constructed, and their Au doping concentrations were 1.6%, 3.1%, and 4.7%, respectively, which were close to the corresponding Au doping concentrations of the abovementioned experimental samples. For the electronic structure calculations, the exchange-correlation potentials among electrons were corrected by local density approximation, and the interaction between ions and electrons was described by ultrasoft pseudopotential. The plane-wave cut-off energy was set at 380 eV, and the Brillouin zone sampling mesh parameters for the *k*-point set were 4 × 4 × 4. The total energy and the interaction force between atoms converged to 5 × 10^−6^ eV/atom and 0.1 eV/nm, respectively, and the internal stress and the displacement were less than 2 × 10^4^ kPa and 5 × 10^−5^ nm, respectively.

## 3. Results and Discussions

### 3.1. SEE Performances of Au-Doped MgO Films

**Figure 1:**
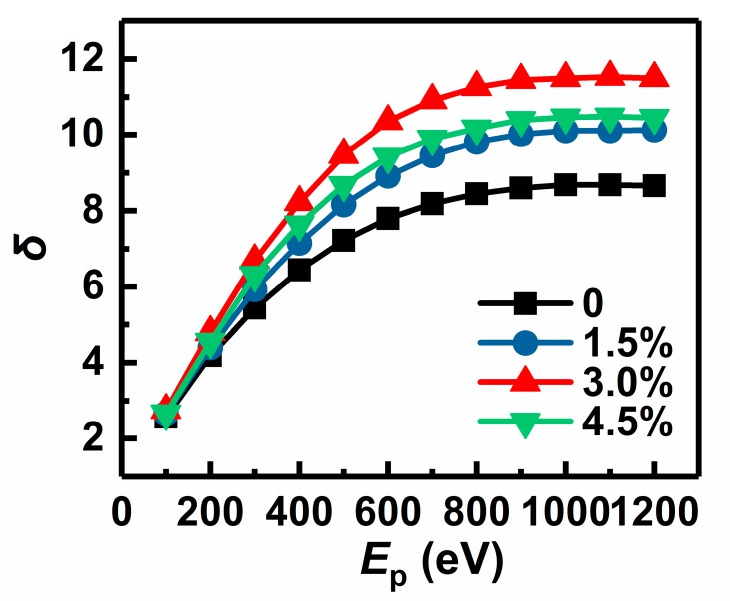
*δ*–*E*_p_ curves of an undoped MgO film sample and three Au-doped MgO film samples with doping concentrations of 1.5%, 3.0%, and 4.5%.

Figure 1 showed the dependences of the SEE coefficients of the undoped MgO film sample and three Au-doped MgO film samples with *x* of 1.5%, 3.0%, and 4.5% upon the primary electron energy. It is obviously seen from Figure 1 that for every MgO film sample, its SEE coefficient has a rapid increase initially and then tends to be saturated as *E*_p_ rises, which conforms to the general SEE law of solid materials. It should be noted that the SEE coefficients at various *E*_p_ values of every Au-doped MgO film sample are higher than those of the undoped one, and the SEE coefficient at every fixed *E*_p_ value takes on a tendency of increasing firstly and then decreasing with the increase of Au doping concentration. Among these four film samples, the Au-doped MgO film sample with *x* of 3.0% has the highest SEE coefficient and its maximum *δ* value reaches 11.5—an increase of 32.2% in comparison with the undoped one. 

**Figure 2:**
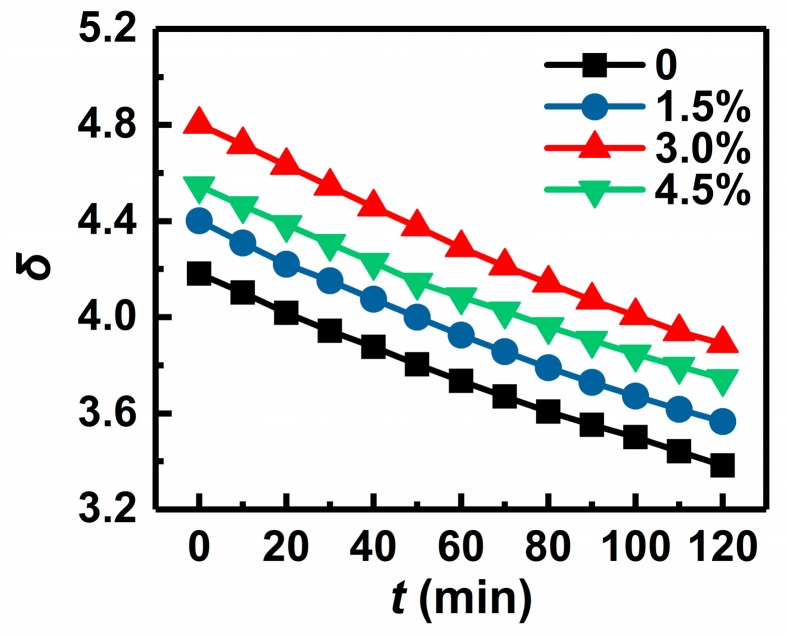
*δ*–*t* curves of an undoped MgO film sample and three Au-doped MgO film samples with doping concentrations of 1.5%, 3.0%, and 4.5%.

In the application of vacuum electronic devices, secondary electron emitters are required to keep a relatively high SEE coefficient for as long as possible under continuous electron bombardment. In order to evaluate the SEE stability of the Au-doped MgO film samples with different doping concentrations, their SEE coefficient variations with time (*t*) of electron bombardment at *E*_p_ of 200 eV were measured, and their *δ*–*t* curves are shown in Figure 2. During the continuous electron bombardment, every Au-doped MgO film maintains a higher SEE coefficient than the undoped one throughout, and among the three Au-doped MgO film samples, the sample with *x* of 3.0% has the highest SEE coefficient. Through calculation, the 2 h SEE degradation rates of the Au-doped MgO film samples with *x* of 0, 1.5%, 3.0%, and 4.5% are 19.1%, 19.0%, 19.0%, and 17.7%, respectively, which shows that the SEE degradation is retarded with the increase of the Au doping concentration. It can be concluded that Au doping in MgO film can not only improve the SEE coefficients at various *E*_p_ values, but also slow down the SEE degradation under continuous electron bombardment. The Au-doped MgO film sample with *x* of 3.0% is considered to have the best SEE performance, because it has the highest SEE coefficient and is speculated to maintain its relatively high SEE coefficient for the longest time under continuous electron bombardment, which better meets the demands of high-gain and long-lifetime vacuum electronic devices.

### 3.2. Surface Morphologies of Au-Doped MgO Films

**Figure 3:**
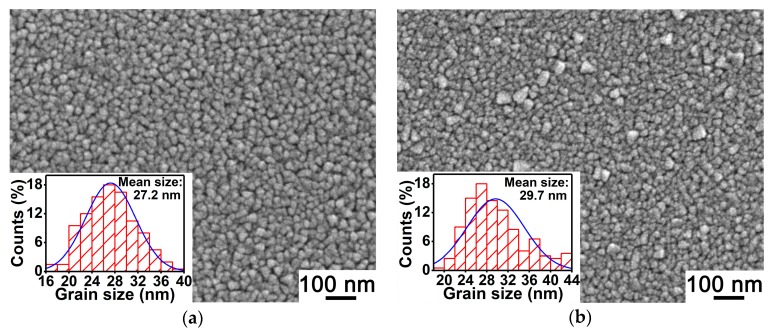

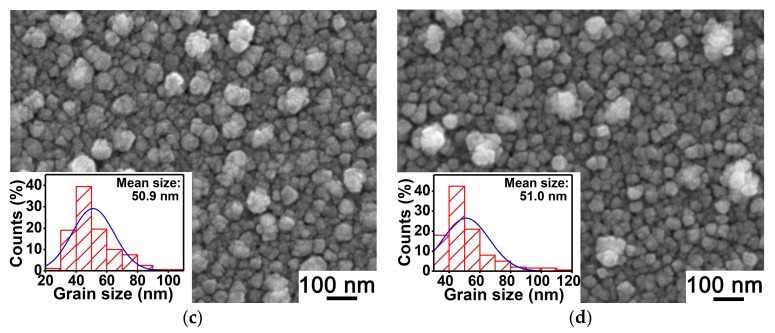
SEM micrographs and MgO grain size probability histograms of (**a**) an undoped MgO film sample and three Au-doped MgO film samples with doping concentrations of (**b**) 1.5%, (**c**) 3.0%, and (**d**) 4.5%.

**Figure 4:**
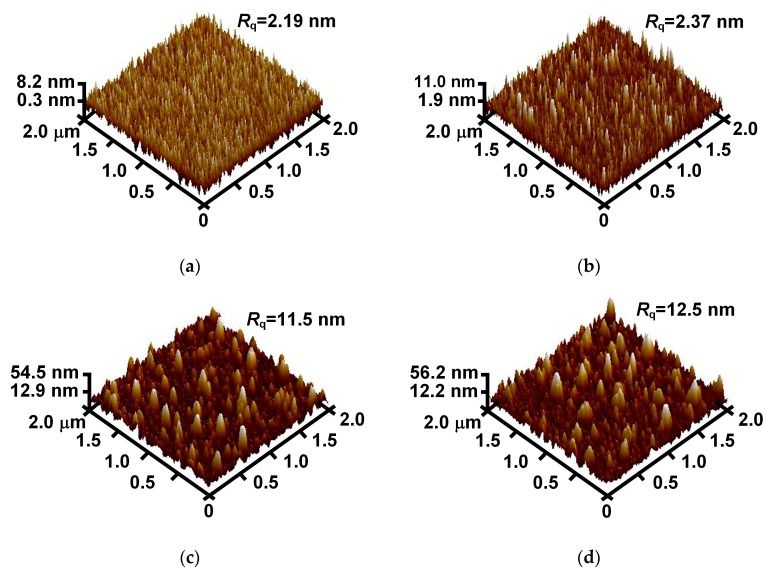
AFM images of (**a**) an undoped MgO film sample and three Au-doped MgO film samples with doping concentrations of (**b**) 1.5%, (**c**) 3.0%, and (**d**) 4.5%.

As surface morphology has a great impact on the SEE of a thin film material [22], SEM micrographs of the abovementioned four MgO film samples were observed and are shown in Figure 3 for studying the effect of Au doping on the SEE performance of MgO film. As shown in Figure 3, the mean grain sizes of Au-doped MgO film samples with *x* of 0, 1.5%, 3.0%, and 4.5% are 27.2 nm, 29.7 nm, 50.9 nm, and 51.0nm, respectively, which reflects that the MgO grain size enlarges with the increase of the Au doping concentration. It is known to us that the MgO and Au crystals have the same face-centered cubic structure and similar lattice constants (0.421 nm for MgO and 0.408 nm for Au). Thus, Au doping in MgO film is conducive to the size enlargement of MgO grains due to the lattice matching of MgO and Au crystals. According to this result, we consider that the increase of SEE coefficient induced by Au doping described in Figure 1 has a close relationship with the MgO grain enlargement. The reduction of grain boundary caused by the enlargement of MgO grains, which decreases the scattering suffered by both the primary electrons injected into the film and the internal secondary electrons moving to the film surface, results in the improvement of the SEE coefficient. However, it should be noted that in comparison with the doped film with *x* of 3.0%, the Au-doped MgO film sample with *x* of 4.5% has a similar grain size, but its SEE coefficients are greatly lower. In order to find out the reason for this phenomenon, the surface roughnesses of these four MgO film samples were measured, and the AFM images are shown in Figure 4.

As shown in Figure 4, the root-mean-square roughness (*R*_q_) of the MgO film rises with the increase of the Au doping concentration. The increase of surface roughness has a negative effect on the SEE, because the emitted secondary electrons from a rough surface may be recaptured by a locally raised surface [23]. According to the SEM and AFM images of the samples with *x* of 0, 1.5%, and 3.0%, as the Au doping concentration increases, the film surface changes to be rougher, while the SEE coefficient becomes higher. Thus, it can be concluded that in the case of large changes in the surface morphology, the grain size enlargement has a greater effect than does the surface roughness increase on the SEE. In addition, compared with the doped film with *x* of 3.0%, the sample with *x* of 4.5% has a similar grain size but a rougher surface, leading to its lower SEE coefficient.

### 3.3. Electronic Structures of Au-Doped MgO Films

For the purpose of further exploring the Au doping effect on the SEE performance of MgO film, the electronic structures of an undoped MgO crystal and three Au-doped MgO crystals with different doping concentrations were obtained by first-principle calculations. Generally, the lower work function of solid materials promotes the generation and escape of internal secondary electrons [24]. As shown in a previous report, MgO films prepared by reactive sputtering generally exhibited the (200) and (220) crystal planes [25]. Thus, the work functions of the (200) and (220) crystal planes of four Au-doped MgO crystals with *x* of 0, 1.6%, 3.1%, and 4.7% were obtained by first-principle calculations, as shown in Table 1. Every Au-doped MgO crystal has obviously reduced work functions of both the (200) and (220) crystal planes in comparison with the undoped one. Additionally, both the work functions of these two crystal planes firstly decrease and then increase as the Au doping concentration rises, and the Au-doped MgO crystal with *x* of 1.6% has the lowest work functions. Thus, the work function reduction induced by Au doping can also lead to the SEE coefficient improvement mentioned in Figure 1.

On the basis of above investigations, the SEE coefficient variation of the Au-doped MgO film is closely connected with the changes in MgO grain size, surface roughness, and work function. Through the comparison between the undoped and Au-doped MgO film, the SEE coefficient increase of MgO film induced by Au doping mainly results from the enlargement of the MgO grain size and the reduction of the work function. With the Au doping concentration changing from 1.5% to 3.0%, the grain size enlargement of the Au-doped MgO film is so great that it leads to SEE enhancement, even though the surface roughness and work function increase. As the Au doping concentration increases from 3.0% to 4.5%, since there is little change in the grain size, so the rougher surface and increased work functions of the Au-doped MgO film are the two main reasons for the reduction of the SEE coefficient.

**Figure 5:**
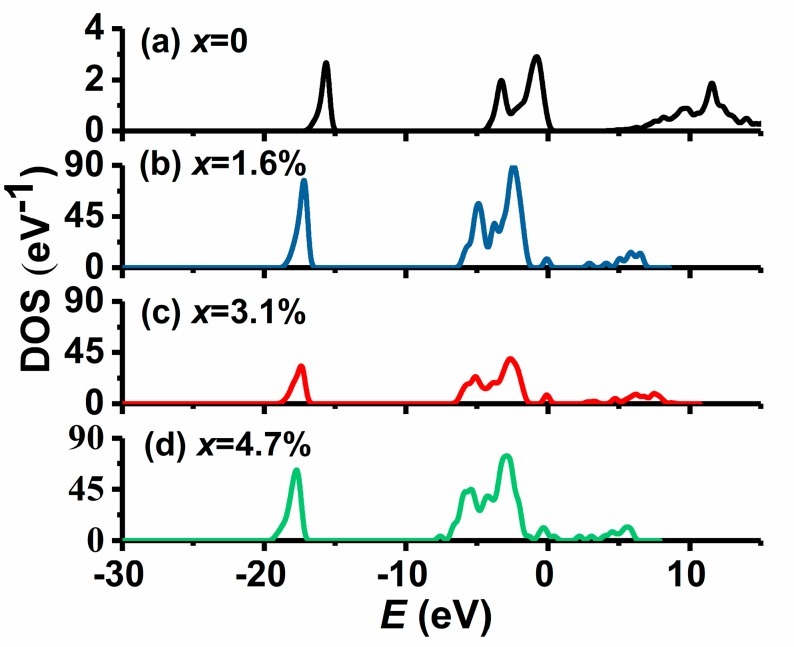
Total densities of electronic states (DOS) of (**a**) an undoped MgO film sample and three Au-doped MgO film samples with *x* of (**b**) 1.6%, (**c**) 3.1%, and (**d**) 4.7%.

Table 2 and Figure 5 show the band gaps and total densities of electronic states (DOS) of the four MgO crystals with different Au doping concentrations based on the respective first-principle calculations. It can be seen from Table 2 that the band gaps of Au-doped MgO crystals with *x* of 0, 1.6%, 3.1%, and 4.7% are 4.76 eV, 2.47 eV, 2.26 eV, and 1.96 eV, respectively, which indicates that the band gap of MgO crystal has an obvious reduction caused by Au doping and it narrows with the increase of the Au doping concentration. It can be seen from Figure 5 that the Au doping leads to the formation of an impurity energy level near the top of the valence band. In addition, both the valence band and the conduction band move to lower-energy positions because of the contribution of Au-5d, and the moving distance of the conduction band is further, which leads to the band gap narrowing of Au-doped MgO crystal.

### 3.4. Electrical Conductivities of Au-Doped MgO Films

**Figure 6:**
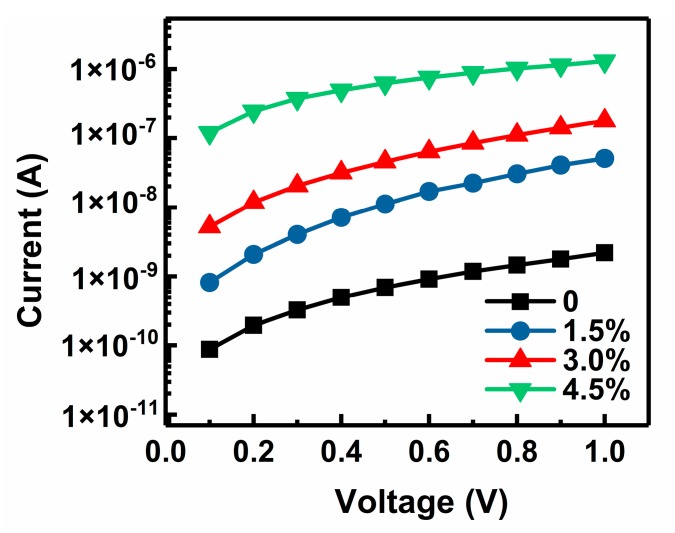
*I*–*V* curves of an undoped MgO film sample and three Au-doped MgO film samples with doping concentrations of 1.5%, 3.0%, and 4.5%.

For an SEE film material, electrical conductivity is an important factor in affecting the SEE degradation property. Good electrical conductivity helps the electrons from the substrate easily migrate through the film and reach the surface to neutralize the positive charges, which can reduce the negative effect of surface charging on the SEE under continuous electron bombardment [15]. Thus, the *I*–*V* curves of an undoped MgO film sample and three Au-doped MgO film samples with different doping concentrations were measured and are shown in Figure 6. It can be evidently seen that the electrical conductivity of Au-doped MgO film rises with the increase of Au doping concentration due to the high conductivity and good chemical stability of Au. In addition, the band gap narrowing of MgO film caused by Au doping described in Table 2 is perhaps another important reason for this improvement of electrical conductivity. Since MgO film is an insulator, surface charging occurs under continuous electron bombardment, which makes the SEE coefficient gradually decrease. The 2 h SEE degradation rate of MgO film decreases with the increase of the Au doping concentration described in Figure 2, although a higher SEE coefficient can result in more serious SEE degradation due to a faster charging process. This phenomenon is likely to be connected with the electrical conductivity improvement induced by Au doping. As the electrical conductivity of the MgO film is improved, the surface charging effect is effectively suppressed; thereby, the SEE coefficient degrades more slowly. 

## 4. Conclusions

In this work, the SEE mechanism of Au-doped MgO film was investigated through surface analysis, first-principle calculation, and electrical characteristic measurement. According to the experimental results, the enhancement of SEE performance is mainly connected with the enlargement of MgO grain size, the reduction of surface work function, and the improvement of electrical conductivity of MgO films caused by Au doping. With the doping concentration of Au-doped MgO film changing from 1.5% to 3.0%, the MgO grain size enlargement has a dominant impact on the increase of SEE coefficient, although the surface roughness and work function rise. However, as the Au doping concentration increases further and reaches 4.5%, the increases of both surface roughness and work function lead to the reduction of the SEE coefficient, since there is little change in the MgO grain size. As to the SEE degradation property of Au-doped MgO film, the improvement of electrical conductivity can effectively suppress the surface charging effect and thereby slow down the SEE degradation under continuous electron bombardment.

## Figures and Tables

**Table 1:** Work functions of the (200) and (220) crystal planes of an undoped MgO crystal and three Au-doped MgO crystals with doping concentrations of 1.6%, 3.1%, and 4.7%.

Au Doping Concentration (%)	Work Function (eV)	
	(200) Crystal Plane	(220) Crystal Plane
0	5.32	5.38
1.6	1.86	2.97
3.1	1.92	3.25
4.7	2.22	3.52

**Table 2:** Band gaps of an undoped MgO crystal and three Au-doped MgO crystals with doping concentrations of 1.6%, 3.1%, and 4.7%.

Au Doping Concentration (%)	Band Gap (eV)
0	4.76
1.6	2.47
3.1	2.26
4.7	1.96
